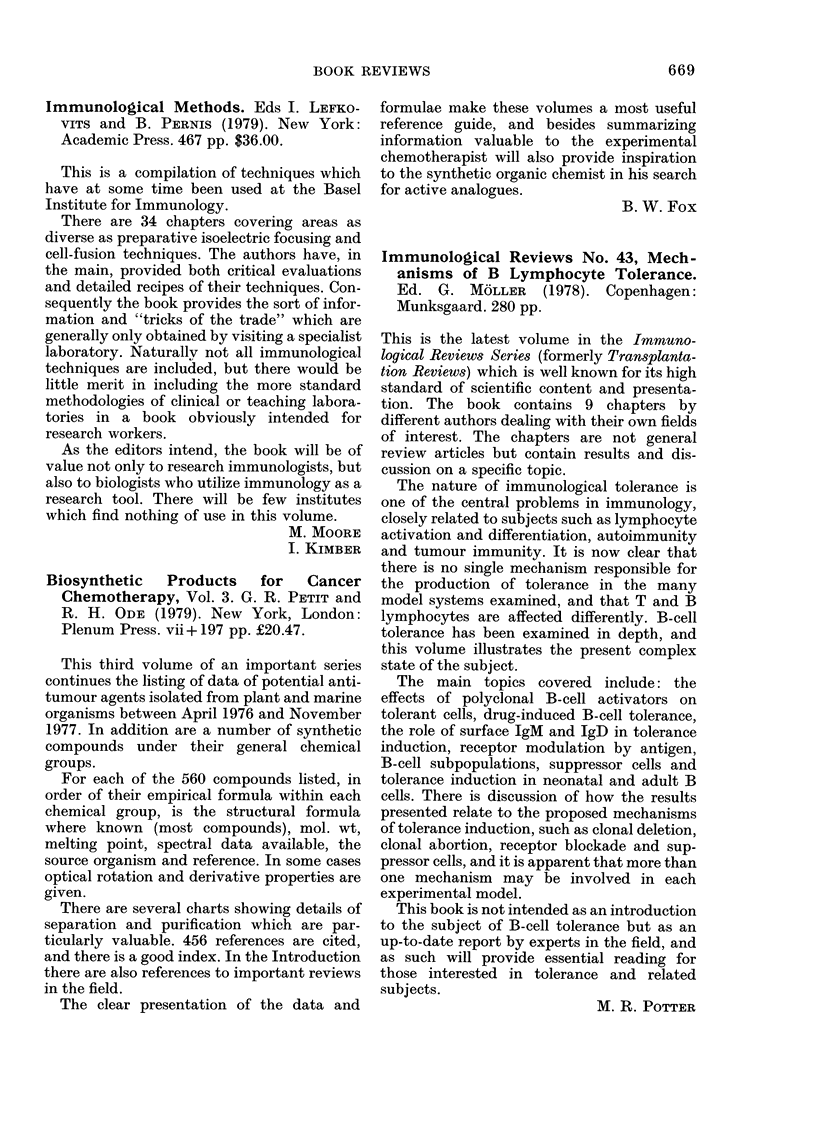# Immunological Methods

**Published:** 1979-10

**Authors:** M. Moore, I. Kimber


**Immunological Methods.** Eds I. LEFKOVITS and B. PERNIS (1979). New York: Academic Press. 467 pp. $36.00.

This is a compilation of techniques which have at some time been used at the Basel Institute for Immunology.

There are 34 chapters covering areas as diverse as preparative isoelectric focusing and cell-fusion techniques. The authors have, in the main, provided both critical evaluations and detailed recipes of their techniques. Consequently the book provides the sort of information and "tricks of the trade" which are generally only obtained by visiting a specialist laboratory. Naturally not all immunological techniques are included, but there would be little merit in including the more standard methodologies of clinical or teaching laboratories in a book obviously intended for research workers.

As the editors intend, the book will be of value not only to research immunologists, but also to biologists who utilize immunology as a research tool. There will be few institutes which find nothing of use in this volume.

M. MOORE
I. KIMBER